# A Dynamic Database of Microarray-Characterized Cell Lines with Various Cytogenetic and Genomic Backgrounds

**DOI:** 10.1534/g3.113.006577

**Published:** 2013-07-01

**Authors:** Zhenya Tang, Dorit S. Berlin, Lorraine Toji, Gokce A. Toruner, Christine Beiswanger, Shashikant Kulkarni, Christa L. Martin, Beverly S. Emanuel, Michael Christman, Norman P. Gerry

**Affiliations:** * Coriell Institute for Medical Research, Camden, New Jersey 08103; † Institute of Genomic Medicine, UMDNJ-NJ Medical School, Newark, New Jersey 07103; ‡ Cytogenomics and Molecular Pathology, Department of Pathology and Immunology, Washington University School of Medicine, St. Louis, Missouri 63110; § Department of Human Genetics, Emory University, Atlanta, Georgia 30322; ** Department of Pediatrics, Children’s Hospital of Philadelphia, and the Perelman School of Medicine at the University of Pennsylvania, Philadelphia, Pennsylvania 19014

**Keywords:** karyotype, FISH, chromosomal microarray analysis, ISCN 2013

## Abstract

The Human Genetic Cell Repository sponsored by the National Institute of General Medical Sciences (NIGMS) contains more than 11,000 cell lines and DNA samples collected from numerous individuals. All of these cell lines and DNA samples are categorized into several collections representing a variety of disease states, chromosomal abnormalities, heritable diseases, distinct human populations, and apparently healthy individuals. Many of these cell lines have previously been studied with detailed conventional cytogenetic analyses, including G-banded karyotyping and fluorescence *in situ* hybridization. This work was conducted by investigators at submitting institutions and scientists at Coriell Institute for Medical Research, where the NIGMS Repository is hosted. Recently, approximately 900 cell lines, mostly chosen from the Chromosomal Aberrations and Heritable Diseases collections, have been further characterized in detail at the Coriell Institute using the Affymetrix Genome-Wide Human SNP Array 6.0 to detect copy number variations and copy number neutral loss of heterozygosity. A database containing detailed cytogenetic and genomic information for these cell lines has been constructed and is freely available through several sources, such as the NIGMS Repository website and the University of California at Santa Cruz Genome Browser. As additional cell lines are analyzed and subsequently added into it, the database will be maintained dynamically.

The National Institute of General Medical Sciences (NIGMS) established the Human Genetic Cell Repository (NIGMS Repository) 40 years ago. The NIGMS Repository is maintained by the Coriell Institute for Medical Research (Coriell Institute) in Camden, New Jersey. To date, more than 11,000 unique samples have been donated by different individuals for the production of cell lines and genomic DNA. These samples are distributed among several collections and represent a variety of chromosomal abnormalities, heritable diseases, numerous distinct human populations, and apparently healthy individuals. All of the samples distributed through the NIGMS Repository have been intensively analyzed by the investigators who submitted the samples originally and/or scientists at the Coriell Institute. As part of its mission to update/supplement the phenotypic information associated with the NIGMS Repository samples, the Coriell Institute utilizes the latest cytogenetic and genomic techniques to analyze samples.

Recently, microarray-based genomic copy number analysis, also called chromosomal microarray analysis (CMA), has become a widely performed genomic diagnostic test (Emanuel and Saitta 2007; Lee *et al.* 2007; Stankiewicz and Beaudet 2007). CMA has several potential advantages over classical cytogenetic techniques, including high resolution, time-savings/cost-effectiveness, and *de novo* analysis without previous phenotypic information. In 2007, the annual volume of CMA tests performed clinically was more than 10,000 in the United States alone (Bejjani and Shaffer 2006; Cheung *et al.*, 2005; Lee *et al.* 2007). In 2010, a panel of experts from International Standard Cytogenomic Array Consortium recommended that CMA testing be applied as the first-tier clinical diagnostic test for individuals with developmental disabilities/intellectual disabilities, autism spectrum disorders, congenital anomalies, or dysmorphic features in place of conventional G-banded karyotype analysis (Miller *et al.* 2010). About the same time, the American College of Medical Genetics and Genomics (ACMG) has issued a series of statement and practice guidelines regarding the clinical use of CMA, including design and performance expectation of the CMA platforms, interpretation, and reporting of the CMA results (Kearney *et al.* 2011a,b; Manning and Hudgins 2010). Up to date, a large number of laboratories have already adapted CMA testing in clinical diagnostics, and many more are interested in applying it as part of standard of care in the near future. Many clinical laboratories and research institutes use NIGMS Repository cell lines and/or DNA samples, particularly samples from the Chromosomal Aberrations and Heritable Diseases collections, as standards or reference materials to develop and validate their CMA assays. To better serve the scientific community, a CMA assay, the Affymetrix Genome-Wide Human SNP Array 6.0 (SNP Array 6.0; Affymetrix, Santa Clara, CA), has been used to further characterize the NIGMS Repository cell lines. To date, approximately 900 cell lines have been analyzed on the SNP Array 6.0 for copy number variations (CNVs) and long contiguous stretches of homozygosity or absence of heterozygosity (AOH), which is also referred to as copy number-neutral loss of heterozygosity as labeled in some figures generated by the ChAS software. We describe herein the construction of a database containing detailed cytogenetic and genomic information for all of these cell lines.

## Composition of the Dataset

In 2010, 716 samples derived from 697 individuals from NIGMS Repository collections were analyzed for CNVs using the SNP Array 6.0. The majority of these samples were chosen from the Chromosomal Aberrations and Heritable Diseases collections. The resulting data, intensity data files (CEL files), and genotype data files (CHP files), along with a limited set of phenotypic information (*e.g.*, gender, age at time of sample collection, and name of diagnosed disease or category of aberration), were deposited in the Database of Genotypes and Phenotypes (dbGaP) under the project name “Genotyping NIGMS Chromosomal Aberration and Inherited Disorder Samples” (Study Accession ID: phs000269.v1.p1). Access to the data is available through dbGaP’s Authorized Access mechanism (https://dbgap.ncbi.nlm.nih.gov/aa/wga.cgi?page=login). Recently, more than 200 additional NIGMS Repository cell lines have been analyzed with the SNP Array 6.0. A large fraction of these additional lines are newly recruited samples from submitters around the world and represent new collections for diseases or disease spectrums not previously held by the Repository, *e.g.*, monosomy 1p36 syndrome, congenital muscular dystrophies, and chromosome 15q11-q13 duplication syndrome, among others. The new CEL files generated from these new cell lines will be available in the dbGaP as well.

All data files (CEL files) were analyzed in Genotyping Console software (Affymetrix). The Contrast QC and MAPD metrics generated by the software were used for quality control purposes. Values of 0.4 and 0.35, respectively, were used as cutoffs for high-quality data. For reporting purposes, the copy number and AOH files (CNCHP files) produced by the Genotyping Console software were subsequently analyzed using Chromosome Analysis Suite (ChAS) software (Affymetrix). All CEL files have been re-analyzed with Nexus 6.0 software (BioDiscovery, El Segundo, CA) with the same parameters described below to confirm all the CNV calls included in this dataset.

The parameters used to call a CNV (loss or gain) in the majority of the cell lines were a segment size ≥100 kb consisting of at least 10 contiguous markers (CNV-only probes) and a confidence score of 95. The minimum parameters used to call a region of AOH were a segment size ≥5 Mb consisting of at least 10 contiguous markers (SNP-only probes) and a confidence score of 95. In addition to analysis using the above default parameters, some samples with a known small pathogenic deletion or duplication previously detected by other methods (*e.g.*, polymerase chain reaction, multiplex ligation-dependent probe amplification, DNA sequencing) were reanalyzed with new parameters (*e.g.*, ≥ 25 kb consisting of at least 10 contiguous markers and a confidence score of 95 for CNVs) that would permit detection of segments of <100 kb. To minimize the possibility of missing a potential pathogenic abnormality, all CNVs (≥25 kb) involving one or more of the genes described in the Online Mendelian Inheritance in Man (OMIM) Morbid Map were reported, even when, according to the clinical information, they were detected in apparently healthy individuals. This is necessary to point out that some of the CNVs, many of which have a size of 25 −100 kb but involve one or more OMIM Morbid Map genes, may not have been further confirmed by other methods at Coriell Institute. The primary goal of this analysis was to extract as much copy number information as possible from each cell line, regardless of clinical pathogenicity. Therefore, the parameters used to generate the database would not necessarily be suitable for clinical diagnostic purposes. Clinical scientists who wish to reanalyze the CEL files should refer to the guidelines issued by the ACMG for the appropriate parameters to be used (Kearney *et al.* 2011a,b; Manning and Hudgins 2010).

All CNV as well as AOH calls were manually checked, whereas the information of probe coverage, allele difference, and segmental duplication at the region where a call is primarily made were considered. Finally, of 879 cell lines at this moment, a total of 6588 CNV calls with an average size of 285 kb covered by an average of 1617 CNV markers and 202 AOH calls (no including the X chromosome) (Rehder *et al.* 2013) with an average size of 11 Mb covered by an average of 3014 SNP-only markers has been made (data not included). Detailed information on each cell line, including CMA results, is available in the NIGMS Repository online catalog and can be openly accessed. The CMA results have been presented in three ways.

First, critical CNVs in each cell line have been reported using the International System for Human Cytogenetic Nomenclature (ISCN). The term “critical CNVs” refers to all chromosomal abnormalities detected or confirmed by G-banded karyotype and/or fluorescence *in situ* hybridization (FISH) analyses, as well as those subtle CNVs (≥25 kb) which have not been detected by conventional methods, but involve one or more OMIM genes as outlined previously. AOH has been detected in many cell lines in this dataset. Examples of AOH in the dataset include AOH affecting one or more segments of a chromosome; a whole chromosome or even the whole genome of a cell line. In general, the clinical significance of the majority of AOH, especially the AOH only affecting chromosomal segment(s) detected in this study, is unknown. The detected AOH regions have not been further confirmed by other methods, such as methylation testing. Therefore, the majority of AOH information has not been integrated into the ISCN reports except in special cases, *e.g.*, GM01225, in which AOH was detected on 14 segments involving 9 different chromosomes (no including the X chromosome) (Rehder *et al.* 2013) of a total size of about 280 Mb (Figure 6); GM11496 with a diagnosis of uniparental disomy of chromosome 7, UPD (7); GM20409 with a diagnosis of UPD (15); GM07489 and GM16810, in which AOH was detected on the whole genome; and other Prader-Willi syndrome (PWS) and Angelman syndrome cell lines, where the UPD of 15q11.2-q13 has been confirmed by submitters using a methylation assay previously. To avoid misleading investigators, unconfirmed AOH information is not disclosed at this moment, but detailed AOH information of each cell line can be provided at individual request. Investigators are encouraged and expected to exchange their own CMA test results on cell lines that they did or are going to choose from this dataset with scientists at Coriell Institute so that a set of confirmed AOH can be published in our online catalog and become accessible to the community in the near future.

**Figure 1:**
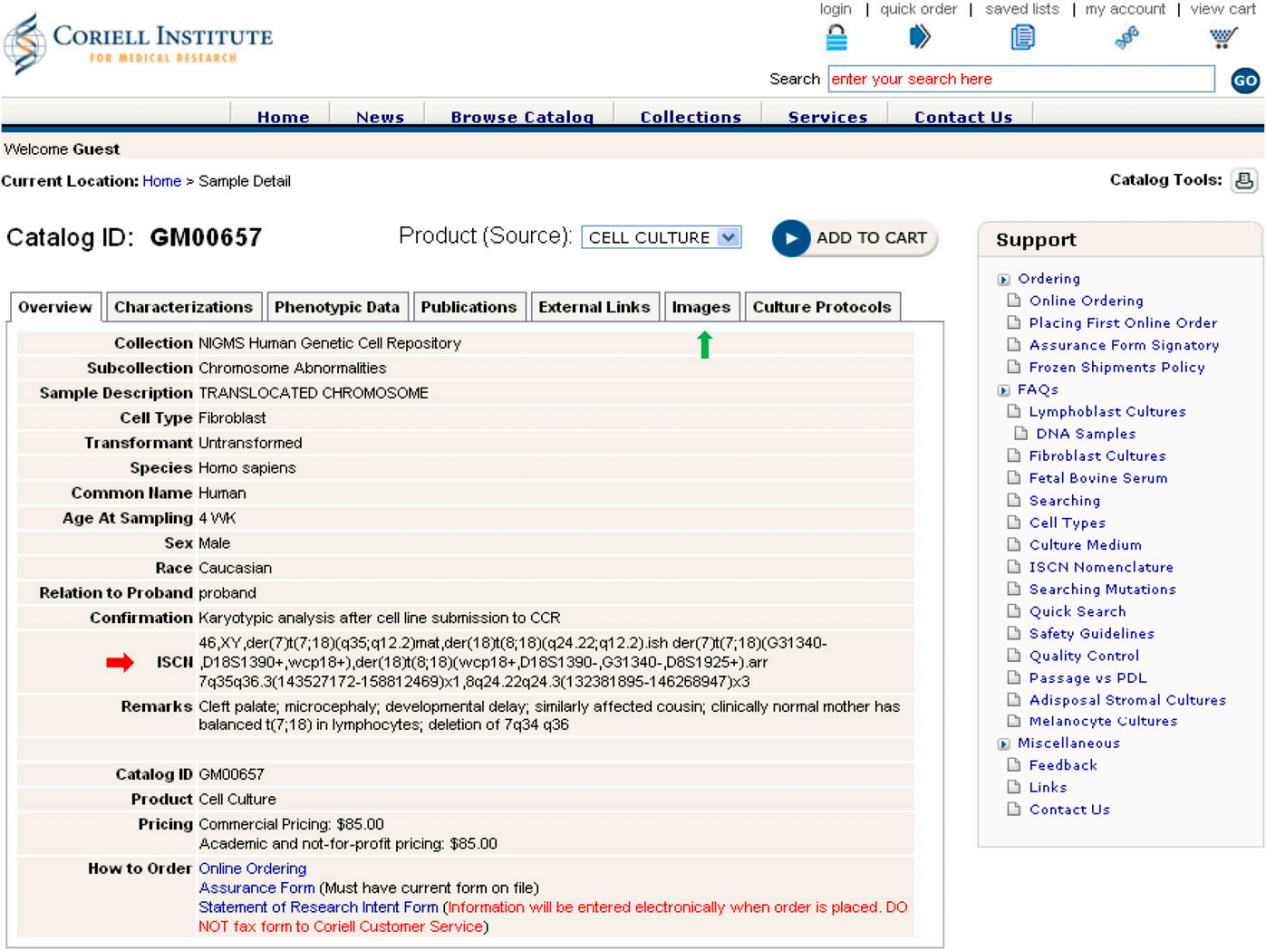
An example of a case presentation in the NIGMS Repository online catalog (cell line GM00657). The red arrow indicates the complete ISCN nomenclature of this cell line. The green arrow indicates links to representative images. Other information, *e.g.*, phenotype data, publication, etc., can be accessed by clicking on related icons on the webpage.

**Figure 2:**
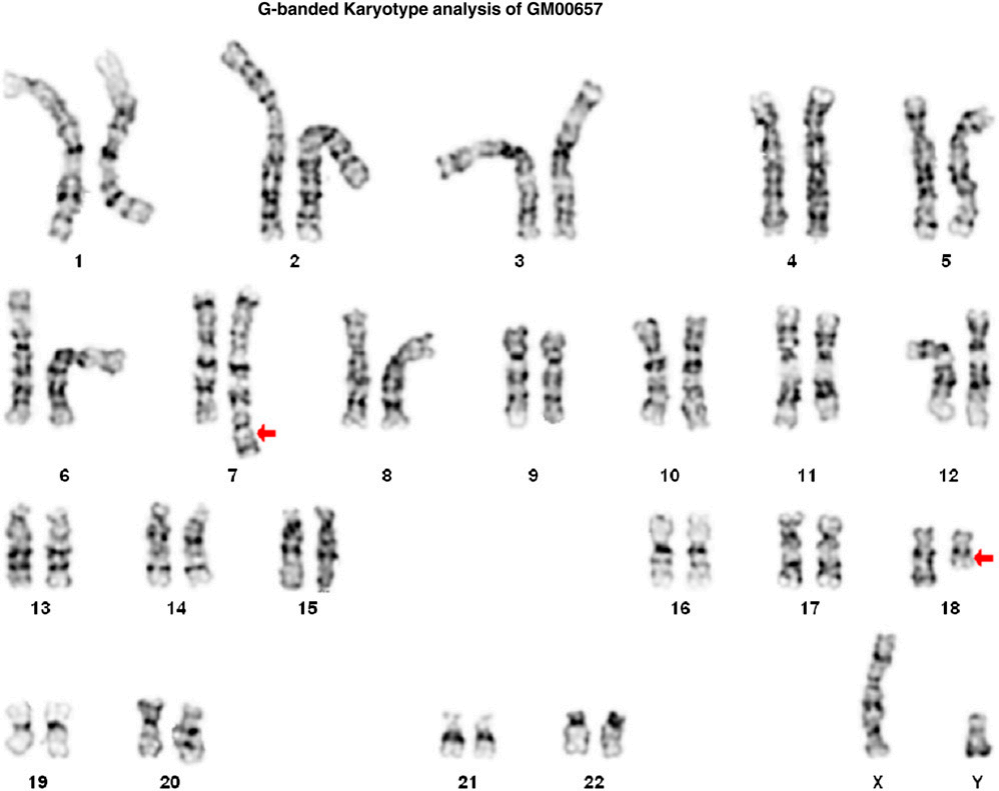
Example of G-banded karyotype analysis of GM00657. Red arrows indicate two abnormal chromosomes, 7 and 18, respectively. Based on the karyotype image, this case was originally considered as a t(7;18) with potentially microdeletion on chromosomes 7, 18, or both. No involvement of the chromosome 8 has been documented at that time.

**Figure 3:**
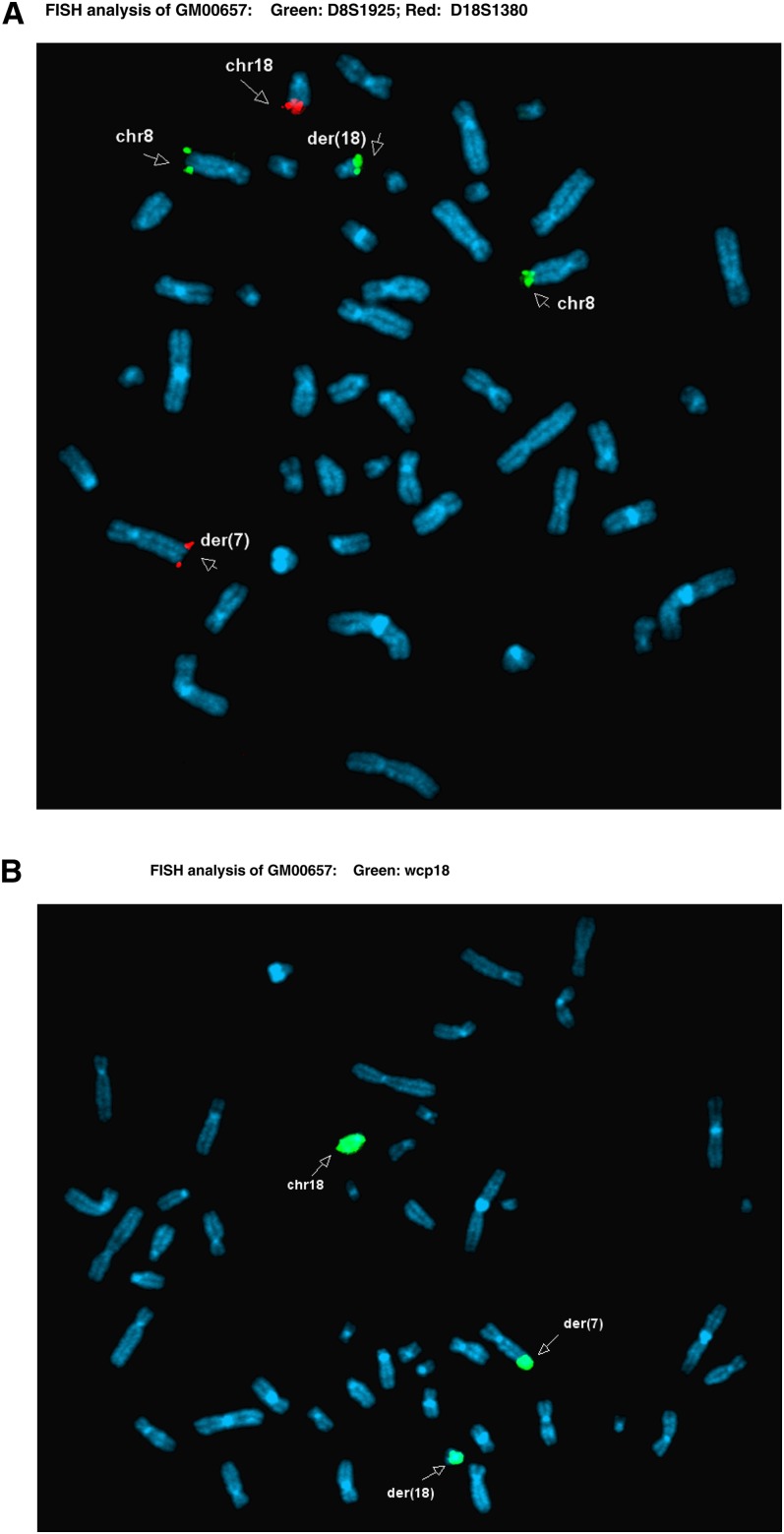
Examples of FISH studies of GM00657. (A) Subtelomere probes for long arm of chromosome 8 (8q) (D8S1925, green color) and long arm of chromosome 18 (18qtel) (D18S1380, red color) were applied to this study. Green hybridization signals for 8qtel have been detected in two normal chromosomes 8 (labeled as chr8) as well as the abnormal chromosome 18, labeled as der(18). This extra green hybridization signal on der(18)indicates a possible gain of 8qtel as well as a structural rearrangement between chromosomes 8 and 18. Red hybridization signals for 18qtel have been detected in the normal chromosome 18 (labeled as chr18) as well as the abnormal chromosome 7, labeled as der(7), but not in the abnormal der(18), indicating possibly no loss of 18qtel, but instead, a translocation involving chromosomes 7 and 18. (B) Whole chromosome paint 18 was applied in this study. In addition to both normal and abnormal chromosomes 18 (labeled as chr18 and der(18), respectively), the distal q arm of the abnormal chromosome 7, der(7), has been painted, further confirming a structural rearrangement between chromosomes 7 and 18, most likely causing a deletion of terminal q arm of the der(7).

**Figure 4:**
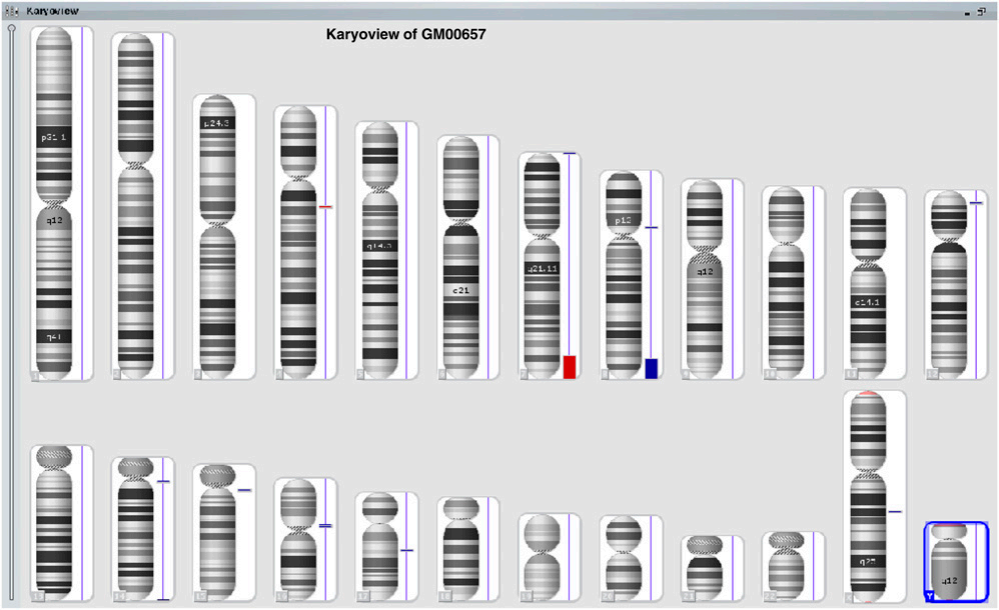
Example of Karyoview of CMA analysis of GM00657. The karyoview presents a diagram of all CNVs (red bar for loss; blue bar for gain) and AOH (purple bar, if present) on the whole genome of the cell line tested. The size of each bar usually reflects the size of the CNV and/or AOH proportionally. The major CNVs in this case are the loss of distal 7q and the gain of distal 8q. Although the chromosomes 18 are involved in structural rearrangement with both chromosomes 7 and 8 as detected by karyotype and FISH analyses, but CMA analysis did not detect a CNV on the chromosomes 18.

**Figure 5:**
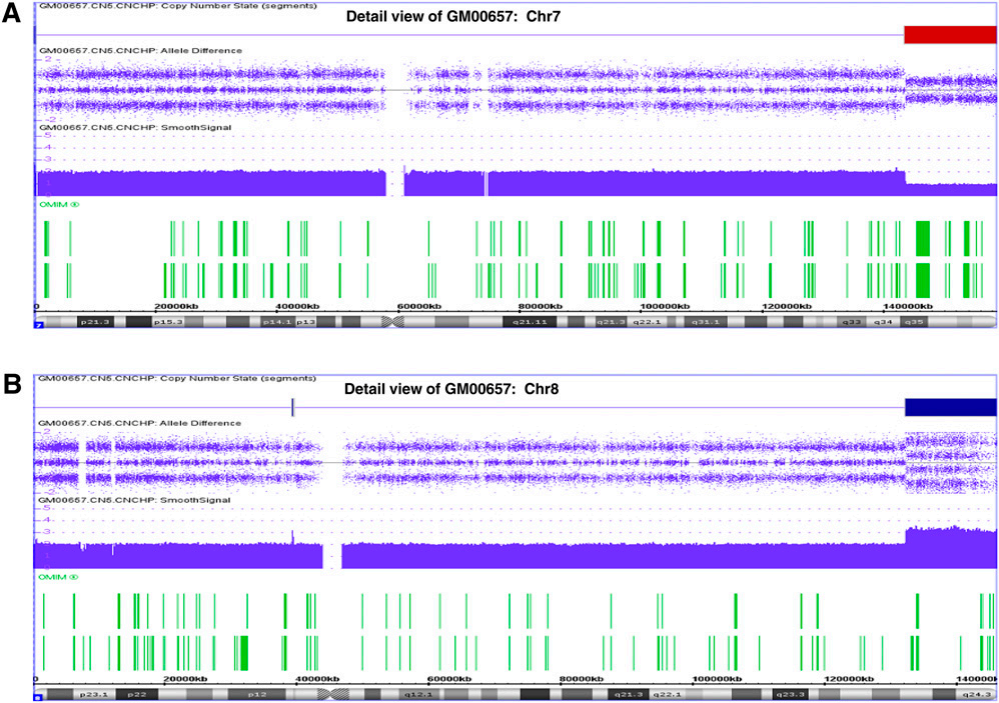
Example of detail or chromosomal view of CMA analysis of GM00657, presenting the specific abnormalities at chromosomal levels. (A) Detail view of chromosome 7 with an obvious deletion of distal 7q; (B) Detail view of chromosome 8 with an obvious gain of distal 8q. In addition to Copy Number State, Allele Difference, Smooth Signals, and OMIM genes shown here, other information, *e.g.*, Log2 ratio, AOH state, FISH probes, segmental duplication, non-OMIM genes, etc., can be presented in the detail view as well.

**Figure 6:**
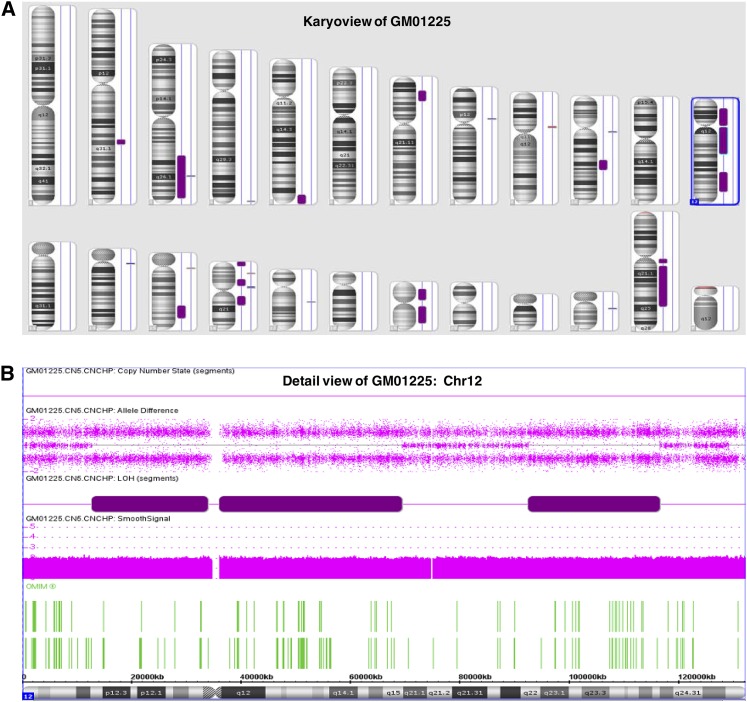
Examples of images of copy number neutral AOH presented in the online catalog. (A) Karyoview of the cell line GM01225 showing AOH (purple bars next to each chromosome ideogram) and CNVs detected. (B) Detail view of chromosome 12 (Chr12) indicating AOH involving three segments through the whole chromosomes 12.

Through the aforementioned NIGMS Repository online catalog, researchers can find all relevant genomic analyses for each cell line. Within the ISCN entry on the Overview tab, all results from G-banded karyotype, FISH, and/or CMA analyses have been presented according to the ISCN 2013 guidelines (Figure 1, cell line GM00657 as an example). Representative karyotype and FISH images demonstrating chromosomal abnormalities detected in a cell line can be obtained by clicking on the Images tab (Figure 2 and Figure 3, cell line GM00657 as an example). One or more images of CMA analysis for each cell line of this dataset may be available and accessible, depending on the numbers of chromosomes and loci affected in the cell line. Typical images available include a “Karyoview,” presenting a diagram of all CNVs (Figure 4, cell line GM00657 as an example) and AOH (Figure 6A, cell line GM01225 as an example) detected in the whole genome, and one or more images of individual chromosomes (“chromosomal view or detail view”) presenting specific abnormalities (Figure 5, cell line GM00657 as an example and Figure 6B, cell line GM01225 as an example).

Second, a spreadsheet including sample ID and short and long ISCN nomenclature describing the chromosomal abnormalities of all cell lines in this database is available both online and as a supplement to this report (*see* Supporting Information, Table S1). Users of the data, especially those comparing it with results obtained by using different platforms than the SNP Array 6.0, are reminded that many factors, including the probe density, analysis parameters, and genome build/annotation files used, will impact the final results.

Third, a total number of 957 critical CNVs (loss or gain) from 602 cell lines has been integrated into a custom track within the University of California at Santa Cruz Genome Browser so far, and they are categorized in the group “Phenotype and Disease Associations” under the name “Coriell CNVs.” This information is readily accessible and searchable by all research scientists.

## Applications of This Database

### Research of interest

The samples in the NIGMS Repository have been used extensively by scientists around the world for a wide variety of research purposes. Publications that use NIGMS Repository samples continually appear in the scientific literature. A list of recent publications can be found on the NIGMS Repository website. Although many investigators may have performed copy number analysis on individual samples of the NIGMS Repository and shared their findings with the Coriell Institute, this database represents the first concerted effort to generate and curate genome-wide copy number data for a large number of samples. Scientists may find numerous mutations in this database that have not been revealed before. For example, a preliminary analysis of the CMA data from approximate 700 cell lines in this database (Z. Tang, D. Berlin and N. Gerry, unpublished data) has resulted in the annotation of 1120 clinically pathogenic and 4470 most likely benign or uncertain clinical significance CNVs (gain or loss) involving all autosomal and sex chromosomes, according to the ACMG guidelines for interpretation and reporting of the CMA results (Kearney *et al.* 2011b). In addition, 165 regions of AOH ≥5 Mb involving all chromosomes except chromosome 20 were detected. More interestingly, approximately 400 of the pathogenic CNVs and almost all of the benign or uncertain clinical significance CNVs had not been previously detected by G-banded karyotype or FISH analysis. Furthermore, the CMA analysis has revealed the AOH status for the first time in most of these cell lines. Therefore, this database provides investigators with detailed cytogenetic and genomic information of each cell line. It can be used as a supplement to existing phenotypic data in the NIGMS Repository online catalog as well as the scientific literature.

### Validation for CMA assays

Although the CMA assay has been recommended as the first-tier clinical diagnostic test for individuals with developmental disabilities or congenital anomalies, the results can vary between individual laboratories (Bejjani and Shaffer 2006; Lee *et al.* 2007; Kaminsky *et al.* 2011; Riggs *et al.* 2012). Some of the sources of this variation include: (1) commercial CMA platforms of different design and content, (2) analysis parameters applied to the raw data to determine the presence of CNVs and/or AOH (*e.g.*, the minimum number of base pairs involved and the minimum number of associated markers), and (3) methods used to validate the CMA assay (*e.g.*, number and types of specimens and their genetic and genomic background). As a result, the development of reference materials that can be widely used for CMA validation has become necessary. The Genetic Testing Reference Materials Coordination Program, led by the Center for Disease Control and Prevention, has been focusing on creating a genomic DNA reference material panel of at least 96 extensively analyzed cell lines containing a broad range of chromosomal abnormalities, *e.g.*, microdeletions and microduplications, subtelomeric abnormalities, UPD, as well as other genetic variations. To date, as part of the Genetic Testing Reference Materials Coordination Program, a panel of 45 cell lines, all chosen from the NIGMS Repository, has been tested with several commercial CMA platforms by their producers (L. Kalman, H. Kearney, L. Conlin, L. Toji, D. Berlin, C. Carmack, J. Gastier-Foster, S. B. Fulmer-Smentek, N. Gerry, L. Jennings, V. Jobanputra, C. Lee, J Leonard, B. Levy, C. Shaw, R. Shippy, S. South, N. Spinner, D. J. Stavropoulos, Z. Tang, H. VanSteenhouse, E. Winn-Deen, D. Wolff, A. Yesupriya, I. Znoyko, and S. Kulkarni). Development of characterized genomic DNA reference material panels for clinical chromosomal microarrays. Presented at the 12th International Congress of Human Genetics/61st Annual Meeting of The American Society of Human Genetics, October 12, 2011, Montreal, Canada). The resulting data have been analyzed by several Cytogenetics experts from various institutes. Currently, this panel of 45 cell lines (“CNVPANEL01”) (Table S2) has been recommended to be applied for developing and validating a CMA assay. It is specifically listed and accessible on the NIGMS Repository website. All the other cell lines documented in this database serve as the pool from which the rest of the specimens for the complete CNV panel will be chosen. This database covers the majority of the most commonly encountered chromosomal conditions in clinical practice, as well as many rarely seen chromosomal abnormalities. Scientists are encouraged to use samples from the CNVPANEL01 or any other cell lines in the database to validate their own CMA assays.

### Reference material panel

Many laboratories focus their testing on a certain disease or disease spectrum. Under these circumstances, a CMA assay is expected to be capable of detecting as many chromosomal conditions related to the disease or disease spectrum as possible. This database can assist laboratories in identifying relevant samples for several diseases and disease spectrums. For example, there are currently 23 cell lines from individuals with PWS and their family members included in this database. The spectrum of variants contained in this subset of cell lines includes microdeletions of various sizes within the 15q11.2-q13 region, as well as examples of both isodisomic and heterodisomic (previously confirmed with methylation tests by the submitters and investigators who used the cell lines) maternal UPD (matUPD) (Z. Tang, D. Berlin, M. Wineburg, A. MacMillan, D. Altamuro, L. Toji, C. Beiswanger, S. Madore, N. Gerry. Mechanism-based Analysis of Human Cell Lines of Prader-Willi Syndrome. Presented at the 62nd Annual Meeting of The American Society of Human Genetics, Date, November 8, 2012 in San Francisco, CA). Many other cell lines in the database also have one or more chromosomal abnormalities involving chromosome 15. Therefore, these cell lines can also be used to build a reference material panel to validate a CMA assay with emphasis on PWS and/or abnormalities on chromosome 15. Other examples of large subsets of samples in the database include, but are not limited to, monosomy 1p36 syndrome, Duchenne muscular dystrophy, Cri-Du-Chat syndrome, and Williams-Beuren syndrome. Many cell lines also have exhibited mosaic karyotypes of different degrees during G-banded karyotype analysis. The mosaic status has also detected by the SNP array 6.0 in some cell lines (Z. Tang, N. Gerry, D. Berlin, A. MacMillan, M. Wineburg, B. A. Frederick, L. Toji, G. A. Toruner, C. Beiswanger. Analysis of 24 Cell Lines of Mosaicism by the Affymetrix Genome-Wide Human SNP Array 6.0. Presented at the 12^th^ International Congress of Human Genetics/61st Annual Meeting of The American Society of Human Genetics, October 12, 2011, Montreal, Canada). These cell lines are good resources to test the ability of a platform to detect mosaic karyotypes.

### Feedback

All investigators are encouraged to provide feedback regarding findings they obtained from analyzing any of the cell lines included in the database, regardless of the assay or platforms used, so that more information about each cell line can be added to the database and shared with the research community. Feedback can be relayed either through the web-based customer services forms or through personal communication with scientists at Coriell Institute by sending an e-mail to NIGMS@coriell.org.

In summary, a database containing genomic information for approximately 900 cell lines, which are mostly chosen from the Chromosomal Aberrations Collection and Heritable Diseases Collection of the NIGMS repository and intensively analyzed by karyotype, FISH, and CMA assays at the Coriell Institute, has been constructed. We believe that this database will serve the clinical and research communities with cell lines and/or DNA samples containing detailed genomic information. This database will be maintained dynamically as additional cell lines are analyzed and subsequently added.

## Supplementary Material

Supporting Information
