# Supplementary material for: A Dynamic Database of Microarray-Characterized Cell Lines with Various Cytogenetic and Genomic Backgrounds
Source: G3 (Bethesda). 2013 Jul 1;3(7):1143–9. doi: 10.1534/g3.113.006577 (PMC3704242; doi:10.1534/g3.113.006577)
Supplement: Supporting Information [file supp_3_7_1143__index.html]

A Dynamic Database of Microarray-Characterized Cell Lines with Various Cytogenetic and Genomic Backgrounds — Supporting Information 

# A Dynamic Database of Microarray-Characterized Cell Lines with Various Cytogenetic and Genomic Backgrounds

## Supporting Information for Tang *et al.*, 2013

**Files in this Data Supplement:**

- Supporting Information - Tables S1 and S2 (PDF, 371 KB)
- Table S2 - Copy Number Variation Panel - CNVPANEL01 (PDF, 312 KB)
- Table S1 - Cell lines and Their Cytogenetic and Genomic Information in the Database (.xlsx, 79 KB)
