# Supplementary material for: A Dynamic Database of Microarray-Characterized Cell Lines with Various Cytogenetic and Genomic Backgrounds
Source: G3 (Bethesda). 2013 Jul 1;3(7):1143–9. doi: 10.1534/g3.113.006577 (PMC3704242; doi:10.1534/g3.113.006577)
Supplement: Supporting Information [file supp_g3.113.006577_TableS2.pdf]

Table S2 Copy Number Variation Panel - CNVPANEL01 (http://ccr.coriell.org/Sections/Collections/NIGMS/cnvpanel01.aspx?pgid=729&coll=GM)

Catalog ID	Cell Type	Description	Short ISCN	Gender	Family
GM01201	B-Lymphocyte	ANEUPLOID CHROMOSOME NUMBER - NON-TRISOMIC	45,XX,-21.arr[hg18] 21q11.2q22.3(13322592-46921373)x1	Female	
GM01416	B-Lymphocyte	XXXX SYNDROME	48,XXXX.arr[hg18](X)x4,(Y)x0	Female	370
GM05067	B-Lymphocyte	ANEUPLOID CHROMOSOME NUMBER - TRISOMY 9	47,XY,+del(9)(q11)mat.arr[hg18] 9p24.3p11.2(36587-44806024)x3	Male	602
GM05966	B-Lymphocyte	DERIVATIVE CHROMOSOME	46,XY,dup(14)(q22q24).arr[hg18] 14q22.2q24.3(54038516-75217413)x3	Male	
GM06226	B-Lymphocyte	TRANSLOCATED CHROMOSOME	46,XY,der(1)t(1;16)(q44;p12)mat.arr[hg18] 1q44(245373155-247190999)x1,16p13.3p12.2(25815-21297471)x3,16p12.1(21853546-22612021)x3	Male	932
GM06870	B-Lymphocyte	ANEUPLOID CHROMOSOME NUMBER - NON-TRISOMIC	47,XX,+i(18)(p10).arr[hg18] 18p11.32p11.21(1543-15391751)x4	Female	966
GM06936	B-Lymphocyte	CHROMOSOME DELETION	46,XX,del(10)(p13).arr[hg18] 10p15.3p13(94427-12918932)x1	Female	974
GM07945	B-Lymphocyte	ADENOSINE DEAMINASE DEFICIENCY WITH NO IMMUNODEFICIENCY	46,XY,del(20)(q12q13.1).arr[hg18] 13q12.11(19701062-19932295)x1,20q11.22q13.12(32961915-44293878)x1	Male	
GM08331	B-Lymphocyte	CHROMOSOME DELETION	46,XY,del(13)(q32q33).arr[hg18] 13q32.1q33.3(96956971-109061570)x1	Male	
GM09102	B-Lymphocyte	CHROMOSOME DELETION	46,XY,del(11)(q23.3).arr[hg18] 11q23.3q25(119996189-134449982)x1,22q11.21(17008946-17386984)x3	Male	
GM09216	B-Lymphocyte	CHROMOSOME DELETION	46,XY,del(2)(p25.1p23).arr[hg18] 2p25.1p23.3(10260988-27005382)x1,4q31.22(145061542-145162384)x1	Male	2124
GM09367	B-Lymphocyte	DUPLICATED CHROMOSOME	46,XX,dup(6)(q21q24).ish dup(6)(q21q24)(wcp6+).arr[hg18] 6q21q24.2(107861056-143105847)x3	Female	

GM09888	B-Lymphocyte	TRICHORHINOPHALANGEAL SYNDROME, TYPE II; TRPS2 (LANGER-GIEDION SYNDROME; LGS)	46,XX,del(8)(q23q24.1).arr[hg18] 1q23.3(159775402-159923110)x3,8q23.1q24.12(107189214-119363784)x1,14q22.1(49891851-50724999)x1,22q11.21(17256416-17420071)x3	Female	
GM10608	B-Lymphocyte	CHROMOSOME DELETION	46,XY,del(20)(p12p11.2).arr[hg18] 20p12.2p11.23(9820603-17979469)x1	Male	
GM10636	B-Lymphocyte	DUPLICATED CHROMOSOME	46,X,dup(X)(p11.4p11.1).ish dup(X)(p11.4p11.1)(STS+,DXZ1++,wcpX+).arr[hg18] Xp11.4p11.1(39693535-57973515)x3,2q13(109819773-111343893)x3	Female	
GM10800	B-Lymphocyte	CHROMOSOME DELETION	46,XY,del(4)(q13.2q22).arr[hg18] 4q13.2q22.2(70096438-95297116)x1	Male	
GM10925	B-Lymphocyte	GREIG CEPHALOPOLYSYNDACTYLY SYNDROME; GCPS	46,XY,del(7)(p14p12).arr[hg18] 7p14.1p11.2(38598541-54681998)x1	Male	1313
GM10985	B-Lymphocyte	CHROMOSOME DELETION	46,XX,del(3)(p25).arr[hg18] 3p26.3p25.3(35333-10305377)x1	Female	
GM10989	B-Lymphocyte	GILLES DE LA TOURETTE SYNDROME; GTS	46,XY,del(9)(p23).ish del(9)(p23)(9ptel30-,D9Z+,wcp9+).arr[hg18] 9p24.3p23(36587-11986831)x1	Male	1316
GM11213	B-Lymphocyte	CHROMOSOME DELETION	46,XX,del(2)(q32.1q33).arr[hg18] 2q32.1q33.2(186818448-204311174)x1	Female	
GM11419	B-Lymphocyte	ANEUPLOID CHROMOSOME NUMBER - NON-TRISOMIC	49,XYYYY.arr[hg18] Xp22.33(109805-2704240)x4,Xq28(154616633-154887040)x4,Yp11.31q12(2712722-27209311)x4,4q31.22(145040166-145270061)x3	Male	1383
GM11672	B-Lymphocyte	CHROMOSOME DELETION	46,XY,del(10)(q11.2q22.1).arr[hg18] 10q11.22q22.2(48962457-75120713)x1	Male	
GM12606	B-Lymphocyte	CHROMOSOME DELETION	47,XY,+del(13)(q21.2).arr[hg18] 13q11q21.2(17943628-59139422)x3	Male	
GM12662	B-Lymphocyte	CHROMOSOME DELETION	46,dup(X)(q28),del(Y)(q11.2).ish del(Y)(q11.2)(DXYS129/DXYS153+,SRY+,DYZ3+,DYZ1+,Z43206+).arr[hg18] Xq28(151659961-154582680)x2,Yq11.223q11.23(22769319-27097245)x0		
GM13019	B-Lymphocyte	TURNER SYNDROME	46,X,idic(X)(p10)[25]/46,X,del(X)(p10)[16]/45,X[9].arr[hg18] Xp22.33p11.1(108464-56912309)x1,Xp11.1q28(62260103-153703648)x2~3	Female	
GM13464	B-Lymphocyte	WILLIAMS-BEUREN SYNDROME; WBS	46,XY.ish del(7)(q11.23q11.23)(ELN-).arr[hg18] 7q11.23(72363697-73780028)x1	Male	

GM13476	B-Lymphocyte	SMITH-MAGENIS SYNDROME; SMS	46,XX.ish del(17)(p11.2p11.2)(D17S29-).arr[hg18] 17p11.2(16704280-20336467)x1	Female	
GM13783	B-Lymphocyte	TRISOMY 21	47,XX,+21.arr[hg18] 21q11.2q22.3(13286389-46887579)x3	Female	
GM14164	B-Lymphocyte	TETRALOGY OF FALLOT	46,XX,del(13)(q13q32).ish del(13)(q13q32)(RB1-,D13S102+).arr[hg18] 13q14.2q32.1(46700085-94512977)x1,22q11.21(17256416-17405213)x3	Female	
GM14485	B-Lymphocyte	INVERTED DUPLICATION DELETION	46,XY,der(8)del(8)(p23.1)dup(8)(p23.1p11.2).ish der(8)del(8)(p23.1)dup(8)(p23.1p11.2)(wcp8+,D8S596-).arr[hg18] 8p23.3p23.1(160290-7213701)x1,8p23.1p11.1(12572787-43719525)x3	Male	
GM14943	B-Lymphocyte	CHROMOSOME DELETION	46,XY,del(2)(q37.1).ish del(2)(q37.1q37.3)(D2S447-,D2Z4-).arr[hg18] 2q37.1q37.3(234941780-242738117)x1	Male	1809
GM15603	B-Lymphocyte	UNIPARENTAL DISOMY CHROMOSOME 8	46,XY.arr[hg18] 8p23.3q24.3(103564-1457732553)x2 hmz	Male	
GM16362	B-Lymphocyte	ANEUPLOID CHROMOSOME NUMBER - TRISOMY	47,XY,+del(22)(q11.2q13.3).ish del(22)(q11.2q13.3)(D22Z1+,TUPLE1-,EWSR1-,ARSA+,D22S1726+).arr[hg18] 22q11.1q11.21(15847411-20903975)x3,22q11.22(20645077-20903975)x1,22q13.2q13.33(41609558-49581309)x3	Male	1925
GM16595	B-Lymphocyte	CRI-DU-CHAT SYNDROME	46,XX,del(5)(p15.2p14).ish del(5)(p15.2p14)(C84C11T7+,D5S721-,D5S23-,EGR1+).arr[hg18] 5p15.2p14.2(8686804-24072399)x1	Female	
GM17867	B-Lymphocyte	XXY SYNDROME; KLINEFELTER SYNDROME	47,XXY.arr[hg18](X)x2,(Y)x1	Male	
GM17942	B-Lymphocyte	DIGEORGE SYNDROME; DGS	46,XY,del(22)(q11.21q11.22).ish del(22)(q11.21q11.22)(TUPLE1-,N85A3+).arr[hg18] 22q11.21(17030682-19792611)x1	Male	
GM20022	B-Lymphocyte	DUPLICATED CHROMOSOME	46,XY,dup(3)(q21q29).ish dup(3)(q21q29)(wcp3+,D3S4560+).arr[hg18] 3q22.2q29(136044785-197137370)x3	Male	
GM20027	B-Lymphocyte	TURNER SYNDROME	45,X.arr[hg18](X)x1,(Y)x0	Female	
GM20556	B-Lymphocyte	ISODICENTRIC CHROMOSOME	47,XY,+idic(15)(q13).ish idic(15)(q13)(D15Z1++,D15S11++,GABRB3++).arr[hg18] Yq11.223q11.23(23920264-27079691)x2,15q11.1q13.3(18276329-30557740)x4	Male	2515

GM21698	B-Lymphocyte	CHROMOSOME DELETION	46,XY,del(6)(q26).ish del(6)(q26)(wcp6+,D62522-).arr[hg18] 6q26q27(162860228-170761408)x1	Male	
GM21699	B-Lymphocyte	CHROMOSOME DELETION	46,XY,der(6)t(3;6)(p26;q26).ish der(6)t(3;6)(p26;q26)(wcp6+,D62522-,D3S4559+).arr[hg18] 3p26.3(35332-580373)x3,6q26q27(163580511-170824447)x1	Male	
GM21887	B-Lymphocyte	ANGELMAN SYNDROME; AS	46,XX,del(15)(q11q13).ish del(15)(q11q13)(D15Z1+,SNRPN-,[D15S10/UBE]-,GABRB3-,PML+).arr[hg18] 15q11.2q13.1(20224751-26500067)x1	Female	
GM22601	B-Lymphocyte	WOLF-HIRSCHHORN SYNDROME; WHS	46,XY,del(4)(p15.2).arr[hg18] 4p16.3p15.2(55665-25591051)x1	Male	
GM22624	B-Lymphocyte	POTOCKI-SHAFFER SYNDROME	46,XX,del(11)(p12p11.2).arr[hg18] 11p12p11.2(40433344-46031324)x1	Female	
GM22991	B-Lymphocyte	CHROMOSOME 1P36 DELETION SYNDROME	46,XX.ish del(1)(p36.32)(CEB108/T7-,SKI-,D1S3739+).arr[hg18] 1p36.32(742429-5215341)x1	Female	
